# The antioxidant activity and metabolomic analysis of the supernatant of *Streptococcus alactolyticus* strain FGM

**DOI:** 10.1038/s41598-024-58933-8

**Published:** 2024-04-10

**Authors:** Xueyan Gu, Heng Wang, Lei Wang, Kang Zhang, Yuhu Tian, Xiaoya Wang, Guowei Xu, Zhiting Guo, Saad Ahmad, Hanyurwumutima Egide, Jiahui Liu, Jianxi Li, Huub F. J. Savelkoul, Jingyan Zhang, Xuezhi Wang

**Affiliations:** 1 Engineering and Technology Research Center of Traditional Chinese Veterinary Medicine of Gansu Province, Lanzhou Institute of Husbandry and Pharmaceutical Sciences, Chinese Academy of Agricultural Sciences, Lanzhou, 730050 China; 2 Cell Biology and Immunology Group, Wageningen University & Research, Wageningen, The Netherlands; 3 Lanzhou Veterinary Research Institute, Chinese Academy of Agricultural Sciences, Lanzhou, 730030 China

**Keywords:** *S. alactolyticus* strain FGM, Antioxidant activity, Metabolomic analysis, Supernatant, Oxidative stress, Cell biology, Microbiology

## Abstract

Strain-specific probiotics can present antioxidant activity and reduce damage caused by oxidation. *Streptococcus alactolyticus* strain FGM (*S. alactolyticus* strain FGM) isolated from the chicken cecum shows potential probiotic properties which have been previously demonstrated. However, the antioxidant properties of *S. alactolyticus* strain FGM remain unknown. In this view, cell-free supernatant (CFS), intact cells (IC) and intracellular extracts (CFE) of strain FGM and 3 strains of *Lactobacillus* (*LAB*) were prepared, and their scavenging capacities against DPPH, hydroxyl radicals and linoleic acid peroxidation inhibitory were compared in this study. The effects of strain FGM cell-free supernatant (FCFS) on NO production, activity of SOD and GSH-Px in RAW264.7 cells and LPS-induced RAW264.7 cells were analyzed. The metabolites in the supernatant were quantitated by N300 Quantitative Metabolome. It was shown that the physicochemical characteristics of CFS to scavenge DPPH, hydroxyl radicals, and linoleic acid peroxidation inhibitory were significantly stronger than that of IC and CFE in the strain FGM (*P* < 0.05), respectively 87.12% ± 1.62, 45.03% ± 1.27, 15.63% ± 1.34. FCFS had a promotional effect on RAW264.7 cells, and significantly elevated SOD and GSH-Px activities in RAW264.7 cells. 25 μL FCFS significantly promoted the proliferation of RAW264.7 cells induced by LPS, increased the activities of SOD and GSH-PX, and decreased the release of NO. Furthermore, among the differential metabolites of FCFS quantified by N300, 12 metabolites were significantly up-regulated, including lactic acid, indole lactic acid, linoleic acid, pyruvic acid etc., many of which are known with antioxidant properties. In conclusion, FCFS had good antioxidant properties and activity, which can be attributed to metabolites produced from strain FGM fermentation. It was further confirmed that *S. alactolyticus* strain FGM and its postbiotic have potential probiotic properties and bright application prospects in livestock and poultry breeding.

## Introduction

Probiotics are defined as “live microorganisms which, when administered in adequate amounts, confer health benefits to the host”^1^. They not only enhance the intestinal mucosal barrier function, improve the balance of intestinal flora, and promote the growth of beneficial bacteria^2,3^. In addition, probiotics can produce various nutrients that participate in human metabolism and hence regulate healthy environment in the intestinal tract^4^. The most commonly used probiotics are bacteria from the genera LAB, *Bifidobacterium,* and *Streptococcus*^5^. Studies have shown that the intake of *L. casei* strains and *L. acidophilus* (LA-5) can increase the quantity of intestinal flora and regulate gut health^6,7^. Consequently, probiotic-based health products play an important role in pharmaceuticals, dietary supplements and functional foods^8^.

Oxygen is extremely important for life activities, but oxidative stress often occurs in the metabolic process that involved oxygen. High oxygen levels can lead to the formation and accumulation of reactive oxygen species (ROS), including superoxide anions (O_2_^−^), hydrogen peroxide (H_2_O_2_) and others^9^, thus causing cell damage, affecting their physiological functions^10^. Prolonged production of ROS is widely acknowledged as a pivotal contributor to the progression of various inflammatory conditions. For instance, LPS originating from the surfaces of Gram-negative bacteria have the capability to directly impair hepatocytes and trigger the activation of Kupffer cells, leading to the production of inflammatory cytokines. This cascade effect can subsequently result in the release of more oxygen species^11,12^. LAB fermentation strains have been found to resist to a variety of reactive oxygen species from the intestinal flora of healthy children^13^. The addition of probiotic complexes in livestock farming has significantly improved growth performance, SOD activity, and reduced levels of malondialdehyde (MDA) and H_2_O_2_ levels in pullets^14^. Have demonstrated effective anti-arthritic activity that was proved by evaluating anti-inflammatory and in vivo antioxidation properties in arthritic rats^15^.

Studies have shown that probiotics play an antioxidant role mainly by scavenging ROS, chelating metals, increasing the levels of antioxidant enzymes and synthesizing antioxidants^16^, and the mechanisms are different among different probiotic strains. Postbiotics are functional bioactive compounds, secreted by live bacteria or released after bacterial lysis^17^, exhibit pleiotropic effects, including immunomodulatory, anti-inflammatory, antioxidant, and anti-cancer properties^18^. It has been reported that extracellular polysaccharides derived from potential probiotic strain possess both antioxidant and antibacterial activities, and can act as prebiotic agents to control pathogenic bacterial biofilm formation^19^. *L. plantarum* T1 CFS isolated from paocai has demonstrated an excellent antioxidative effect in RAW264.7 cells^20^.

In a previous study conducted in our laboratory, *S. alactolyticus* strain FGM was isolated from the contents of the chicken cecum^21,22^. The results suggest that *S*. *alactolyticus*^23^ is an important player in the gut microbiota and contributes to the diet adaptation of giant pandas by primarily engaging in protein metabolism^24^. It was previously demonstrated that polysaccharides from fermented Astragalus membranaceus by *S. alactolyticus* had some similar properties to those from Astragalus membranaceus in terms of its ability to help healing hepatic fibrosis in rats and modulate immunopotentiation of broiler chicken^23^.

However, the oxygen radical absorbance capacity (ORAC), cell antioxidant activity and metabolomics analysis of *S. alactolyticus* strain FGM, have not been reported yet. To explore the potential development value of metabolites derived from this strain, we conducted an evaluated of the ORAC of various components of the FGM strain, using *L. casei*, *L. acidophilus*, and *L. reuteri* as reference points. Additionally, we analyzed the impact of FGM strain components exhibiting higher ORAC values on factors such as NO production, ROS levels, as well as SOD and GSH-PX activities in RAW264.7 cells and LPS-induced RAW264.7 cells. Furthermore, we conducted metabolomic analysis of the strain's supernatant. This comprehensive approach aims to assess the antioxidative potential and metabolic profile of the strain FGM and its metabolites for potential future applications.

## Materials and methods

### Strains and cells

*S. alactolyticus* strain FGM (GenBank accession No. JX435470; China Patent No. 20120141827.5) was derived from the cecum of indigenous chickens, isolated and preserved in the Veterinary Laboratory of Lanzhou Institute of Husbandry and Pharmaceutical Sciences of CAAS. RAW264.7 macrophage cells were obtained from Sunlight Bio-technology (Shanghai, China). The *L. acidophilus* (BNCC, China, No. 185342), *L. reuteri* (BNCC, China, No. 192190) and *L. casei* (BNCC, China, No.134415) were purchased from North Natron link Biotechnology Ltd.

### Strains resuscitation and bacteria fractions preparation

*S. alactolyticus* strain FGM, *L. acidophilus*, *L. reuteri and L. casei* stored at − 80 °C were thawed at 37 °C water bath, inoculated into MRS (Huankai, China) nutrient broth and incubated at 37 °C for 24 h. They were then cultured to a second or third passage for subsequent experiments.

Bacterial fractions were prepared according to the method described in the literature^25^. Briefly the bacterial concentration was adjusted to 1 × 10^8^ cfu/mL, the supernatant was obtained by centrifugation (4500×*g* for 10 min at 4 ℃). After passing through a sterile 0.22 μm pore-size filter unit, a CFS was obtained. The cell pellet was washed three times with 0.01 mmol/L phosphate buffer saline (PBS, pH 7.4) and resuspended in PBS. The cell pellet was adjusted to 1 × 10^8^ cfu/mL to obtain IC. CFE were obtained from the cell suspensions containing 1 × 10^8^ cfu/mL that were subjected to ultrasonic disruption (in ice cold water, ten 5-s strokes and 5-s intervals, 40 min) and centrifuged (12,000×*g* for 10 min at 4 °C) to remove the cell debris.

The RAW264.7 cells were cultured in DMEM (Gibco, USA) supplemented with 10% FBS (Gibco, China), 100 U/mL penicillin, and 100 μg/mL streptomycin (Beyotime, China). The cells were incubated in a humidified atmosphere containing 5% CO_2_ at 37 °C. Cells were passaged every 2 to 3 days upon reaching 70–80% confluency.

### Antioxidant assays of different bacteria fractions

#### DPPH radical scavenging activity (RSA)

The DPPH RSA of different bacteria fractions was assessed using the method referenced in Lin et al.^26^. Briefly, 500 μL of each sample was mixed with 500 μL of DPPH solution and incubated in the dark for 30 min within a 1.5 mL centrifuge tube. For the blank, 500 µL of absolute ethanol was used, while 500 µL of MRS broth or PBS served as controls. After the incubation period, the absorbance of the mixture was measured at 517 nm and DPPH RSA is expressed as follows.

$${\text{DPPH RSA }}\left( \% \right)\, = \,\left[ {{1}\, - \,\left( {{\text{A Sample}}\, - \,{\text{A Control}}} \right)/{\text{A Blank}}} \right]\, \times \,{1}00\% .$$

#### Hydroxyl RSA

The hydroxyl RSA was measured according to the method described by Zhang^27^. The reaction mixture was prepared by combining the following components in a test tube: 1 mL PBS, 0.5 mL of 1,10-phenanthroline ethanol solution (Solarbio, China), 0.5 mL of FeSO_4_, 0.5 mL of the bacterial fraction being tested, 0.5 mL of H_2_O_2_. This mixture was incubated at 37 °C for 90 min and measured at 536 nm. The data was expressed as a blank. 0.5 mL H_2_O_2_ was replaced with 0.5 mL distilled water, and the data was expressed as controls, using 0.5 mL distilled water was replaced with 0.5 mL sample, and the data was expressed as sample. The percentage of resistance to hydroxyl radicals was defined as follows.

$${\text{Hydroxyl RSA }}\left( \% \right)\, = \,\left( {{\text{A Sample}}\, - \,{\text{A Blank}}} \right)/({\text{AControl}}\, - \,{\text{A Blank}})\, \times \,{1}00\% .$$

#### Linoleic acid peroxidation inhibitory activity

1 mL Linoleic acid (Unsaturated fatty acids) which was prepared of 0.1 mL linoleic acid, 0.2 mL Tween 20 (Solarbio, China) and 19.7 mL deionized water. 0.5 mL PBS, 0.2 mL FeSO_4_, 0.2 mL H_2_O_2_ and 0.5 mL of different bacterial fractions were mixed and incubated at 37 °C for 12 h. Blank samples contained either PBS or MRS. 2 mL of the reaction solution was mixed with 0.2 mL TCA (Solarbio, China), 2 mL TBA (Solarbio, China) and 0.2 mL BHT (Solarbio, China), and incubated at 100 °C for 30 min before added 2 mL chloroform. The absorbance value of the extracts was measured at 532 nm^26^. The formula is as follows.

$${\text{Linoleic acid peroxidation inhibitory activity }}\left( \% \right)\, = \,\left( {{1}\, - \,{\text{A Sample}}/{\text{A Blank}}} \right)\, \times \,{1}00\% .$$

### The effects of FCFS in RAW264.7 cells

#### Cell viability assay

The CCK-8 assay (ZETA, USA) was used to evaluate the effect of the FCFS on the growth of RAW264.7 cells. Initially, RAW264.7 cells were seeded into 96-well plates and were cultured for 12 h. Subsequently, the medium was refreshed, and different concentrations of FCFS working solutions were added to the cells for another 12 h. 0.1 μg/mL LPS as a positive control, while RPMI-1640 complete medium (CCM) as a negative control. After discarding the culture supernatant, each well was incubated with CCM containing 10% CCK-8 at 37 ℃ in the dark for 1 h to 1.5 h. The OD values in each well were measured at 450 nm using a microplate reader.

#### NO production

The macrophages were seeded into a 6-well plate (1 × 10^6^ cfu/mL) for at least 12 h. Subsequently, 1 mL CCM along with various concentrations of the different FCFS were added to each well and the plate was incubated for 24 h. Following incubation, the culture medium at 2000 rpm for 20 min to collected supernatants, and cells supernatant were analyzed with the Griess kit (ThermoFisher, USA) according to the manufacturer’s instructions. The NO levels were calculated from the absorbance at 540 nm measured by a microplate reader.

#### ROS activity

The levels of intracellular oxidative stress were determined using ROS assay kit (Beyotime, China). Briefly, RAW264.7 cells pretreated with FCFS were collected, resuspended in freshly prepared serum-free medium containing 10 μmol/L DCFH-DA and incubated at 37 °C in the dark for 20 min. The cells were then harvested and washed with PBS buffer. The absorbance value was measured the using a fluorescence microplate reader at 488 nm excitation wavelength and 525 nm emission wavelength.

#### SOD and GSH-Px activity

RAW264.7 cells were centrifuged and resuspended to achieve a concentration of 1 × 10^6^ cfu/mL, then cultured in 6-well plate for 12 h. Subsequently, 200 μL cell lysis solution was added to each well, which was repeatedly blown to make the cells shed, lyse, and release intracellular proteins, and were centrifuged at 12,000×*g* for 5 min. The resulting supernatant was collected for the assessment of SOD (Beyotime, China) activity and GSH-Px (Beyotime, China) activity.

### The effects of FCFS in LPS induced RAW264.7 cells

#### Cell viability assay

RAW264.7 cells (5 × 10^5^ cfu/mL) were seeded into 96-well plates and cultured for 12 h. The cells were then treated with 25 μL and 125 μL FCFS and the plates were incubated for additional 12 h. After incubation, 100 μL LPS was added for 24 h, and following two washes with PBS, 100 μL CCM was added, and 10 μL CCK-8 reagent was added to each well. After further incubation 1.5 h, the absorbance was measured at a wavelength of 450 nm to determine cell viability.

#### NO production

The release of NO from RAW264.7 cells was measured by the quantifying the stable end product of NO oxidation with the Griess reagent, according to a previous report^28^. The cells were pretreated with 25 μL and 125 μL FCFS and the plates incubated for 12 h. Then 0.5 µg/mL LPS was added to stimulate the cells for 24 h. The supernatant was collected and centrifuged at 2000 rpm for 20 min to remove any cellular debris. Each well was added 80 µL supernatant and 80 µL of both Griess reagent I and II. The absorbance was determined at 540 nm using a microplate reader (Supplementary Information).

#### SOD and GSH-Px activity

RAW264.7 cells were treated with LPS and then harvested for the analysis of antioxidant enzymes. SOD activity and GSH-Px activities were measured using commercial assay kits and the absorbance was measured at 450 nm with a microplate reader, and the corresponding activity was calculated by a formula.

#### Metabolomics analysis of FCFS

The metabolomics analysis of FCFS was carried out using liquid-chromatography-mass spectrometry (LC–MS) technology, specifically on the ACQUITY UPLC Xevo TQ-S platform, to investigate the metabolomic profiles and their biological functions (data from three groups, 5 biological repeats for each)^29,30^. The MS detection process involved the analysis of blank samples (MRS), quality control samples (QC), and experimental samples. To achieve absolute quantitative results for the metabolites present in the samples, chromatographic data analysis was performed using MassLynx V4.1 software. Subsequent multivariate statistical analyses of the metabolites, including principal component analysis (PCA) and partial least squares discriminant analysis (PLS-DA), were conducted to identify differences in metabolic patterns of different groups. Volcano map and KEGG pathway database were employed to annotate the distinct metabolites and to highlight the pathways that exhibited differential metabolite enrichment. This metabolomics study was performed in the laboratory of Beijing Novozymes Technology Co.

### Statistical analysis

Data were expressed as mean ± SD, One-way ANOVA determined differences between groups with the SPSS 26.0 software and GraphPad Prism 6.0. For all the tests, a two-sided P-value of < 0.05 was considered significant.

## Results

### DPPH RSA

DPPH RSA has been attributed to the hydrogen donating ability of antioxidants and is commonly employed in antioxidant assays. IC, CFS and CFE from *S. alactolyticus* strain FGM*, L. acidophilus, L. reuteri* and *L. casei* were prepared. The DPPH RSA of different probiotic components is shown in Table 1. The DPPH RSA of VC was 96.87% ± 1.09. The DPPH RSA of CFS, IC and CFE in the strain FGM was 87.12% ± 1.62, 31.05% ± 8.15 and 24.5% ± 8.14, respectively. Notably, the DPPH RSA of CFS in the strain FGM showed no significant differences compared to that of *L. acidophilus, L. reuteri and L. casei* (*P* > 0.05). However, the DPPH RSA of IC in the strain FGM was significantly higher than that in *L. casei* and *L. acidophilus*. On the other hand, the DPPH RSA of CFE in the strain FGM was notably lower than that in *L. acidophilus* and *L. reuteri*, yet significantly higher than *L. casei* (*P* < 0.05).

**Table 1:** Total DPPH radical scavenging activity assays.

Sample name	DPPH radical scavenging activity (%)			
		CFS	IC	CFE
*S. alactolyticus* FGM		87.12 ± 1.62^ab^	31.05 ± 8.15^a^	24.50 ± 8.14^c^
*L. casei*		94.40 ± 1.05^a^	18.47 ± 3.99^b^	9.99 ± 2.93^d^
*L. acidophilus*		86.98 ± 6.58^ab^	16.24 ± 3.88^b^	68.04 ± 9.88^a^
*L. reuteri*		86.10 ± 2.23^b^	25.87 ± 3.08^ab^	39.13 ± 6.24^b^
Vitamin C	96.87 ± 1.09			

In each column, values followed by different letters are statistically different according to the ANOVA test with the same small letter mean no significant difference (*P* > 0.05), while with different small letters means significant difference (*P* < 0.05). The same as below.

### Hydroxyl RSA

Hydroxyl radicals are most reactive entities among the ROS and are known to cause DNA damage and lipid peroxidation^31^. As shown in Table 2, the hydroxyl RSA of VC was52.22% ± 1.39. The hydroxyl RSA of CFS, IC, and CFE in the strain *S. alactolyticus* FGM was 45.03% ± 1.27, 34.14% ± 2.05 and 8.48% ± 5.16, respectively. Compared with *L. acidophilus*, strain FGM exhibited a significantly lower level of hydroxyl RSA in CFS (*P* < 0.05), but there was no difference difference when compared with *L. reuteri*, and *L. casei* (*P* > 0.05). Regarding the hydrolxyl RSA of IC, strain FGM was significantly higher than that of *L. reuteri* (*P* < 0.05), noticeably lower than that of *L. casei* (P < 0.05), and showed no significant difference from *L. acidophilus* (*P* > 0.05). For the hydroxyl RSA of CFE, strain FGM was significantly lower than that of *L. casei* (*P* < 0.05), but here was no significant difference when compared with *L. acidophilus* and *L. reuteri* (*P* > 0.05).

**Table 2:** Total Hydroxyl radical scavenging activity assays.

Sample name	Hydroxyl radical scavenging activity (%)			
		CFS	IC	CFE
*S. alactolyticus* FGM		45.03 ± 1.27^bc^	34.14 ± 2.05^b^	8.48 ± 5.16^b^
*L. casei*		42.45 ± 0.73^c^	57.47 ± 2.92^a^	17.38 ± 3.90^a^
*L. acidophilus*		56.93 ± 4.01^a^	27.24 ± 4.79^b^	11.27 ± 0.94^ab^
*L. reuteri*		47.04 ± 0.75^b^	18.94 ± 5.30^c^	10.39 ± 1.46^ab^
Vitamin C	52.22 ± 1.39			

### Linoleic acid peroxidation inhibitory activity

Linoleic acid from natural sources has been reported to inhibit lipid peroxidation^32^. As shown in Table 3, the inhibitory activity of the *S. alactolyticus* FGM strain from CFS, IC and CFE was 15.63% ± 1.34, 5.66% ± 1.89 and 14.47% ± 1.09, respectively. Interestingly, the FCFS exhibited the highest inhibitory activity on Linoleic acid peroxidation and was significantly higher than that of *L. casei* and *L. reuteri* (*P* < 0.05), but there was no significant difference when compared with *L. acidophilus* (*P* > 0.05). For the IC form all four strains, there was no significant difference (*P* > 0.05). Comparing with the CFE of the strains, the FGM strain showed an activity that was significantly higher than *L. casei* and *L. acidophilus* (*P* < 0.05), but there was no significant difference with *L. reuteri* (*P* > 0.05).

**Table 3:** Total Linoleic acid peroxidation inhibitory activity assays.

Sample name	Linoleic acid peroxidation inhibitory activity (%)		
	CFS	IC	CFE
*S. alactolyticus* FGM	15.63 ± 1.34^a^	5.66 ± 1.89^a^	14.47 ± 1.09^a^
*L. casei*	6.98 ± 0.81^c^	5.31 ± 0.18^a^	9.31 ± 1.61^b^
*L. acidophilus*	15.21 ± 1.95^a^	4.82 ± 0.40^a^	6.75 ± 2.57^b^
*L. reuteri*	10.39 ± 1.50^b^	4.94 ± 2.39^a^	16.98 ± 1.89^a^

### The effects of FCFS in RAW264.7 cells

#### Cell viability assay

**Figure 1:**
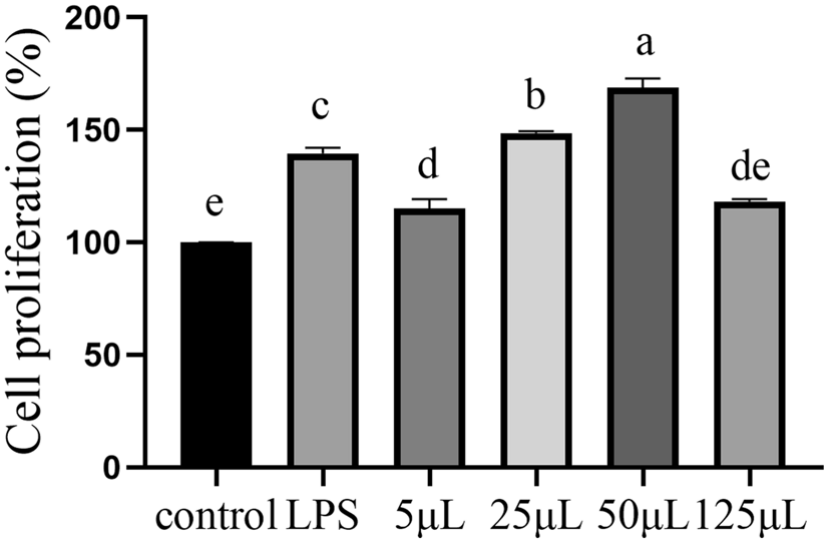
Effects of different concentrations of FCFS on the proliferation of RAW264.7 cells. Effects of different concentrations of FCFS on the proliferation of RAW264.7 cells. In the figure, groups that share the same lowercase letter are not significantly different (*P* > 0.05), whereas groups with different letters are significantly different (*P* < 0.05).

The result of the proliferative capacity of RAW264.7 cells in the FCFS groups and the LPS group are showed in Fig. 1. As the dose of FCFS increased, the cell proliferation rate showed an ascending trend from 5 to 50 μL, but then decreased at 125 μL. The range of 25 μL to 125 μL FCFS, as well as the LPS treatment, significantly promoted cell proliferation in RAW264.7 cells (*P* < 0.05).

### NO production

**Figure 2:**
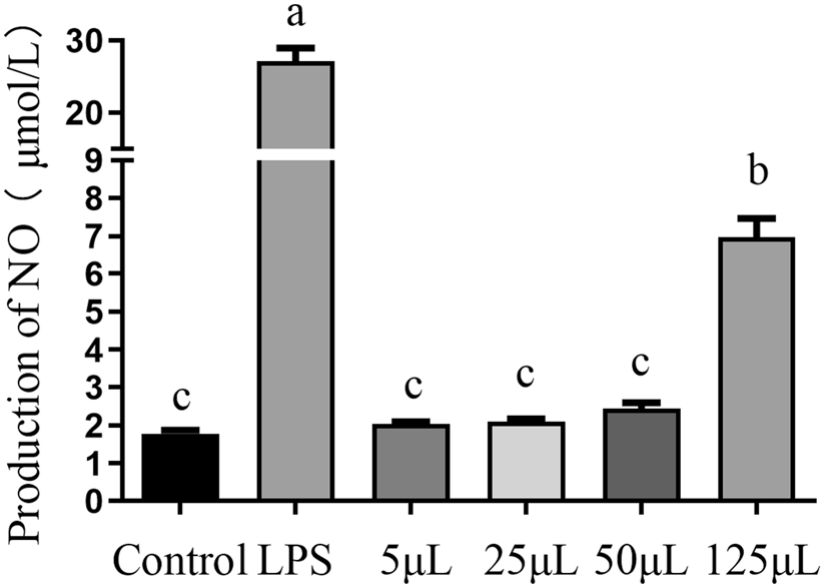
Effects of different concentrations of FCFS on production of NO of RAW264.7 cells. In the figure, groups that share the same lowercase letter are not significantly different (*P* > 0.05), whereas groups with different letters are significantly different (*P* < 0.05).

NO produced during host inflammatory responses is involved in various disorders. The result of No production in RAW264.7 cells stimulated with doses ranging from 5 to 125 μL of FCFS and with 100 μL of LPS are shown in Fig. 2. A clear positive correlation observed between the FCFS dose and cellular NO production, with the group receiving 125 μL FCFS exhibiting significantly higher levels than the control group (*P* < 0.05). Furthermore, the LPS group showed an exceptionally potent effect in inducing NO production compared to the FCFS groups (*P* < 0.01).

### ROS activity

**Figure 3:**
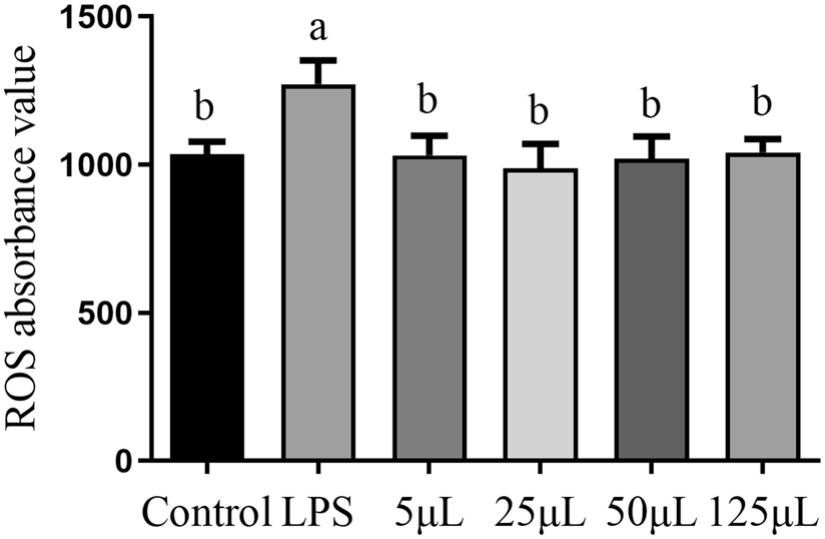
The effects of different concentrations of FCFS on ROS level of RAW264.7 cells. In the figure, groups that share the same lowercase letter are not significantly different (*P* > 0.05), whereas groups with different letters are significantly different (*P* < 0.05).

Excessive ROS can induce oxidative stress and inflammatory responses, serving as one of important indicators of cellular damaged. As shown in Fig. 3, when compared to the control group, the doses of 5 μL, 25 μL, 50 μL, and 125 μL of FCFS did not exhibit a significant effect on the ROS levels in RAW264.7 cells (*P* > 0.05). However, ROS levels in the FCFS groups were notably lower than those observed in the LPS group (*P* < 0.05).

### SOD and GSH-Px activity

**Figure 4:**
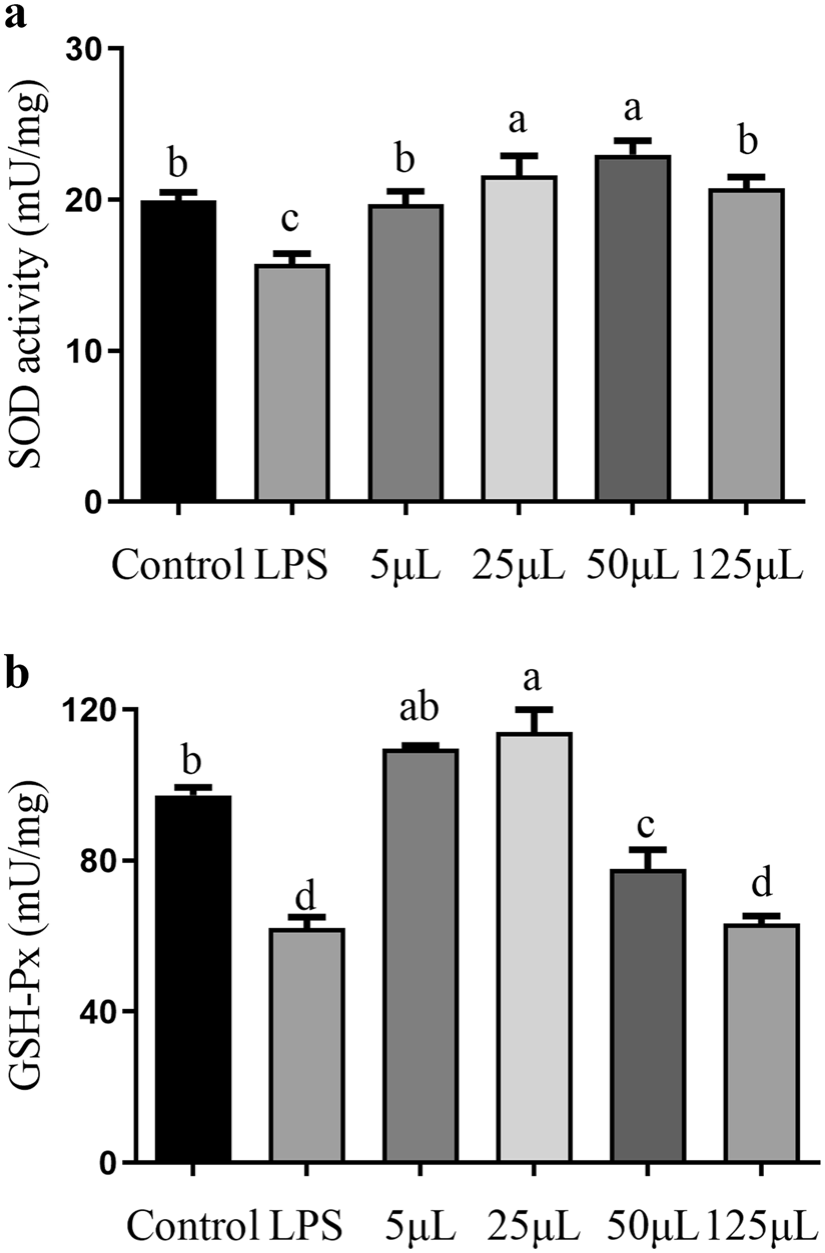
The effects of different concentrations of FCFS on SOD (**a**) and GSH-Px activity of RAW264.7 cells (**b**). In the figure, groups that share the same lowercase letter are not significantly different (*P* > 0.05), whereas groups with different letters are significantly different (*P* < 0.05).

SOD and GSH-Px are both crucial antioxidant enzymes within cells. As presented in Fig. 4, doses of 25 μL and 50 μL of FCFS significantly increased SOD activity compared to the control group (*P* < 0.05). SOD activity showed an ascending trend with increasing FCFS doses from 5 to 50 μL, followed by a decrease at 125 μL. In contrast, SOD activity in the LPS group was significantly lower compared to control group (*P* < 0.05). Regarding the GSH-Px activity, 25 μL of FCFS dose was significantly higher than the control group (*P* < 0.05). However, 50 μL and 125 μL of FCFS doses resulted in significantly lower than the control group (*P* < 0.05), showing a similar effect with LPS group.

### The effects of FCFS in LPS induced RAW264.7 cells

#### Cell viability assay

**Figure 5:**
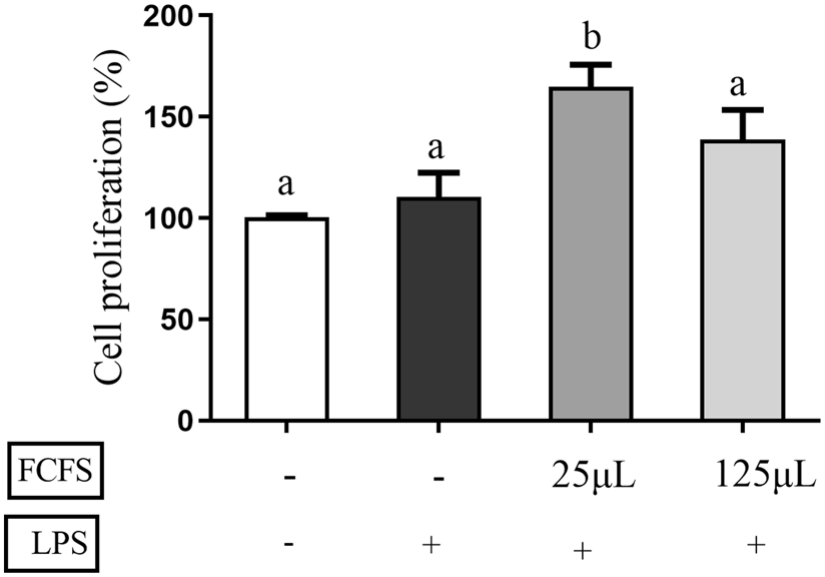
Effects of different concentrations of FCFS on the proliferation of RAW264.7 cells induced by LPS. In the figure, groups that share the same lowercase letter are not significantly different (*P* > 0.05), whereas groups with different letters are significantly different (*P* < 0.05).

A cell viability assay was conducted to evaluate the effects of fermented cell-free supernatant (FCFS) on RAW264.7 cells in an LPS-induced oxidative stress model. As depicted in Fig. 5. a dose of 25 μL FCFS significantly promoted cell proliferation in LPS-induced RAW264.7 cells compared to compared to both the 0.5 μg/mL LPS group and CON group (*P* < 0.05). However, there was no difference with 125 μL CFS (*P* > 0.05).

### NO production

**Figure 6:**
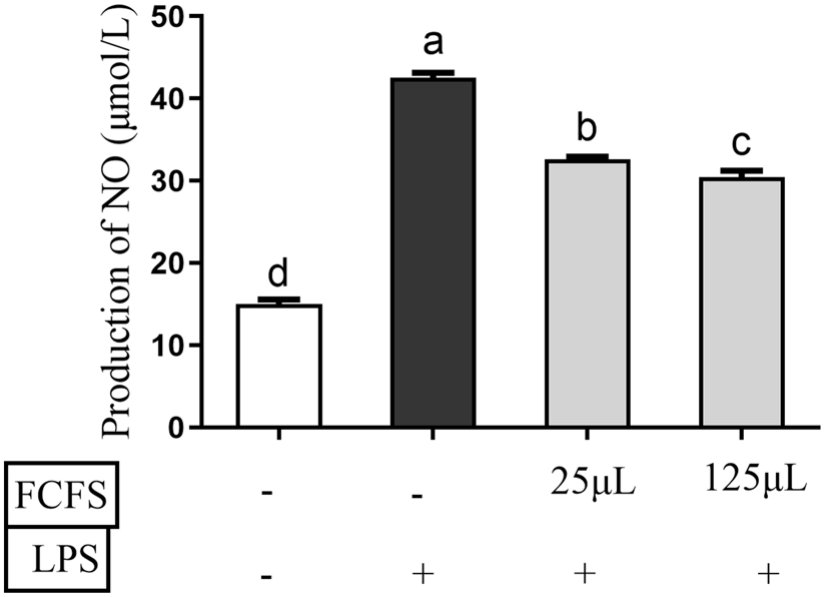
Effects of different concentrations of FCFS on production of NO of RAW264.7 cells induced by LPS. In the figure, groups that share the same lowercase letter are not significantly different (*P* > 0.05), whereas groups with different letters are significantly different (*P* < 0.05).

A previous study reported that different strains demonstrate distinct inhibitory effects on LPS-induced NO synthesis^33^. As shown in Fig. 6, both 25 µL and 125 µL doses of FCFS significantly reduced the concentration of NO in LPS-induced RAW264.7 cells compared to 0.5 μg/mL LPS group (*P* < 0.05). Notably, NO concentration in the group treated with 125 μL FCFS was significantly lower than that in the 25 μL group (*P* < 0.05).

### SOD, and GSH-Px activity

**Figure 7:**
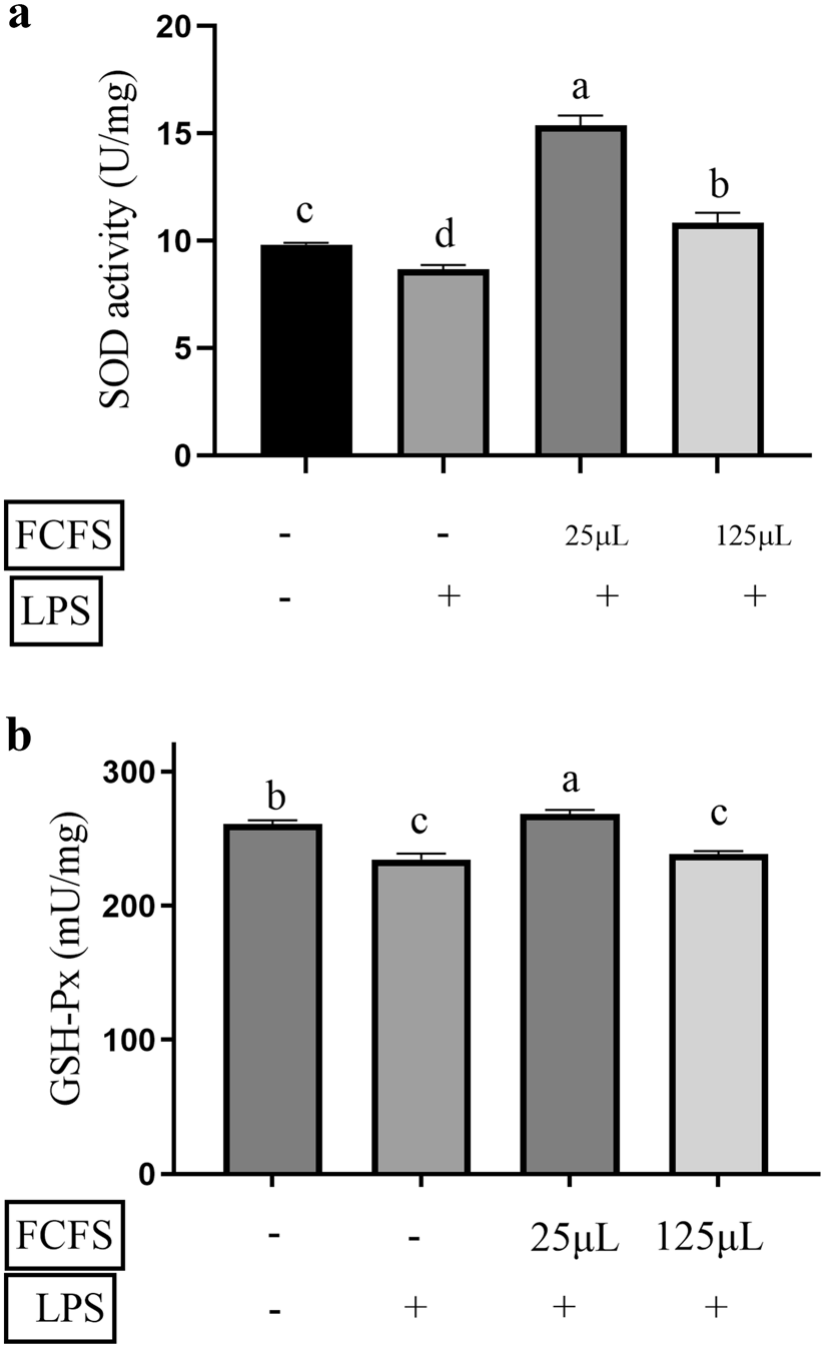
The effects of different concentrations of FCFS on SOD (**a**) and GSH-Px activity of RAW264.7 cells induced by LPS (**b**). In the figure, groups that share the same lowercase letter are not significantly different (*P* > 0.05), whereas groups with different letters are significantly different (*P* < 0.05).

The oxidative indicators including SOD and GSH-Px were assessed to evaluate the anti-oxidative property of FCFS on oxidative stress in LPS-induced RAW264.7 cells. As shown in Fig. 7, treatment with 25 μL FCFS significantly increased the activities of SOD and GSH-Px in LPS-induced RAW264.7 cells compared to 0.5 μg/mL LPS group (*P* < 0.05). However, the activities of SOD and GSH-Px in the group treated with 125 μL FCFS were significantly lower than those in the 25 μL FCFS group (*P* < 0.05).

### Overview of metabolomics differences

#### Screening and analysis of differential metabolites

**Figure 8:**
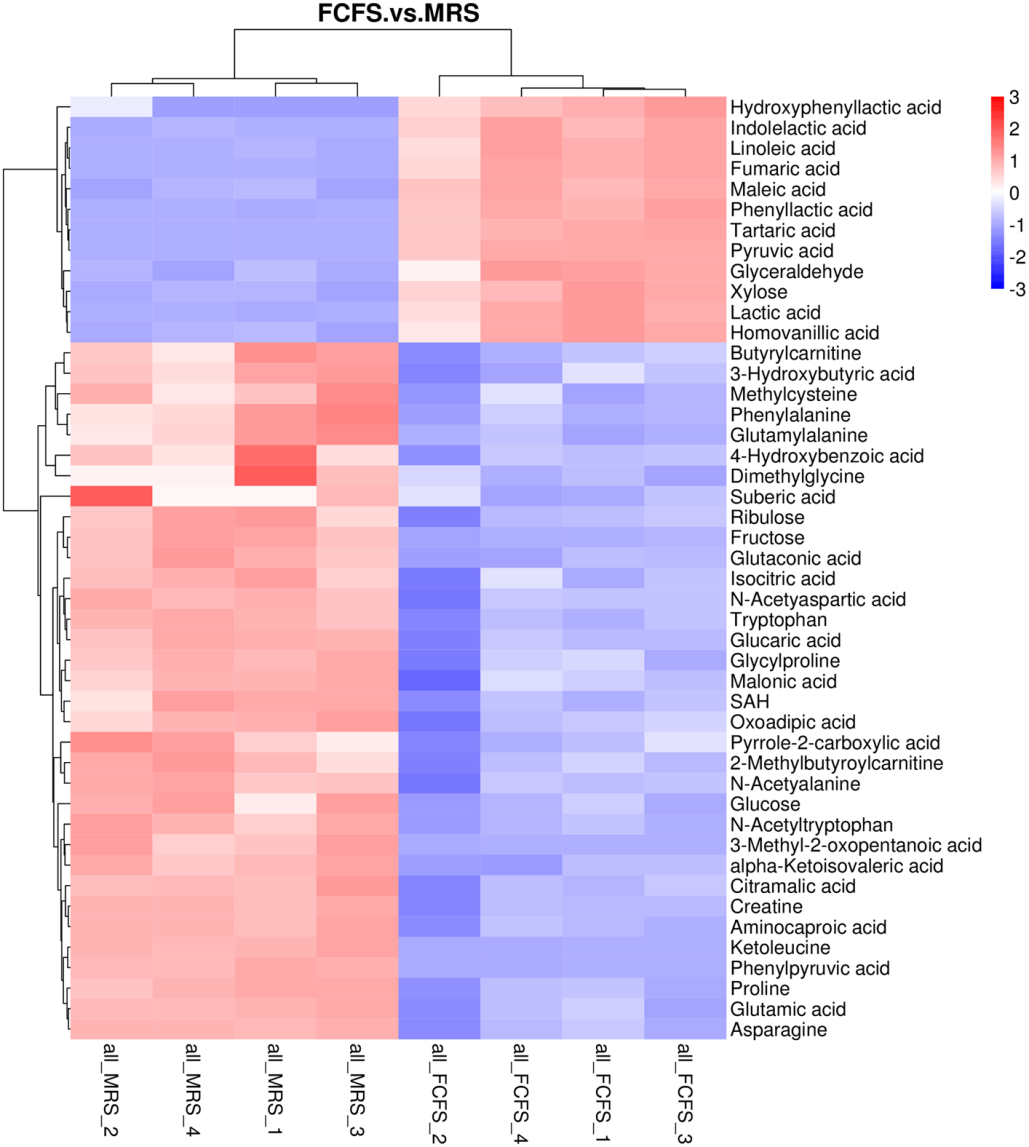
Heat map of FCFS and MRS differential metabolites, the heatmap was created in “clusterProfler” R software package, version 3.4.3. The longitudinal is the clustering of FCFS and MRS, and the transverse is the clustering of metabolites, with shorter cluster branches representing higher similarity.

After data processing, 156 differential metabolites were standardized and analyzed by MassLynx V4.1 software to study cellular metabolites in FGM and MRS. Subsequent screening based on the principles of variable importance projection (VIP) with VIP > 1.0, fold change FC > 1.5 or FC < 0.667, and P < 0.05 in the OPLS-DA model, by means of the identification of 46 differential metabolites through database comparison. The specific differential metabolites are shown in Tables 4 and 5. As shown in Fig. 8, the relative content of these metabolites is represented by variations in color intensity on the Heat map. The trend in the changes of differential metabolites can be broadly categorized into two zones. In the red zone of the upper half corresponding to the FCFS sample group, the metabolites are predominantly Phenols and Indoles, including 2 Phenylpropanoic Acids, 1 Fatty Acids, 3 Carbohydrates, and 4 Organic Acids, all of which are significantly upregulation. Conversely, the red zone in the lower half highlights the differential metabolites mainly found in the MRS sample group.

**Table 4:** Differential metabolites.

Compared samples	Num. of total ident.	Num. of total sig.	Num. of sig.up	Num. of sig.down
FGM vs. MRS	156	46	12	34

**Table 5:** Metabolites with significant differences in FCFS.

Name	Group	FC	P value	VIP	Up down
Fumaric acid	Organic acids	2.969	0.000	1.205	Up
Pyruvic acid	Organic acids	4.798	0.000	1.203	Up
Linoleic acid	Fatty acids	5.234	0.000	1.202	Up
Phenyl lactic acid	Phenyl propanoic acids	4.285	0.000	1.200	Up
Tartaric acid	Carbohydrates	17.078	0.000	1.199	Up
Xylose	Carbohydrates	1.707	0.000	1.199	Up
Lactic acid	Organic acids	17.365	0.000	1.198	Up
Homovanillic acid	Phenols	2.880	0.000	1.196	Up
Indole lactic acid	Indoles	6.620	0.000	1.196	Up
Glyceraldehyde	Carbohydrates	2.751	0.000	1.192	Up
Maleic acid	Organic acids	2.969	0.000	1.186	Up
Hydroxy phenyl lactic acid	Phenyl propanoic acids	2.570	0.009	1.097	Up
Phenyl pyruvic acid	Benzenoids	0.148	0.000	1.186	Down
Ketoleucine	Organic acids	0.215	0.000	1.185	Down
3-Methyl-2-oxopentanoic acid	Organic acids	0.272	0.000	1.181	Down
Fructose	Carbohydrates	0.467	0.000	1.175	Down
Glutaconic acid	Organic acids	0.587	0.000	1.159	Down
*N*-Acetyltryptophan	Amino acids	0.652	0.000	1.157	Down
Alpha-Ketoisovaleric acid	Organic acids	0.373	0.001	1.144	Down
Proline	Amino acids	0.495	0.002	1.138	Down
Creatine	Amino acids	0.658	0.002	1.138	Down
Glutamylalanine	Peptides	0.521	0.001	1.132	Down
Glutamic acid	Amino acids	0.631	0.003	1.131	Down
Tryptophan	Amino acids	0.665	0.002	1.131	Down
Asparagine	Amino acids	0.528	0.004	1.126	Down
Glucaric acid	Carbohydrates	0.660	0.004	1.121	Down
Citramalic acid	Fatty acids	0.647	0.003	1.116	Down
Aminocaproic acid	Amino acids	0.363	0.006	1.111	Down
Phenylalanine	Amino acids	0.604	0.001	1.110	Down
Glucose	Carbohydrates	0.467	0.001	1.110	Down
SAH	Nucleotides	0.559	0.001	1.108	Down
Ribulose	Carbohydrates	0.664	0.002	1.100	Down
*N*-Acetyaspartic acid	Amino acids	0.654	0.009	1.088	Down
2-Methylbutyroylcarnitine	Carnitines	0.645	0.004	1.086	Down
Butyrylcarnitine	Carnitines	0.601	0.001	1.084	Down
*N*-Acetyalanine	Amino acids	0.653	0.008	1.083	Down
Suberic acid	Fatty acids	0.439	0.006	1.083	Down
Methyl cysteine	Amino acids	0.551	0.002	1.076	Down
Glycyl proline	Peptides	0.597	0.012	1.073	Down
3-Hydroxybutyric acid	Organic acids	0.629	0.005	1.071	Down
Dimethylglycine	Amino acids	0.630	0.011	1.070	Down
Oxoadipic acid	Organic acids	0.635	0.009	1.066	Down
4-Hydroxybenzoic acid	Benzoic acids	0.654	0.003	1.060	Down
Isocitric acid	Organic acids	0.580	0.013	1.058	Down
Pyrrole-2-carboxylic acid	Organic acids	0.568	0.006	1.044	Down
Malonic acid	Organic acids	0.636	0.024	1.032	Down

#### PCA and OPLS-DA analysis

**Figure 9:**
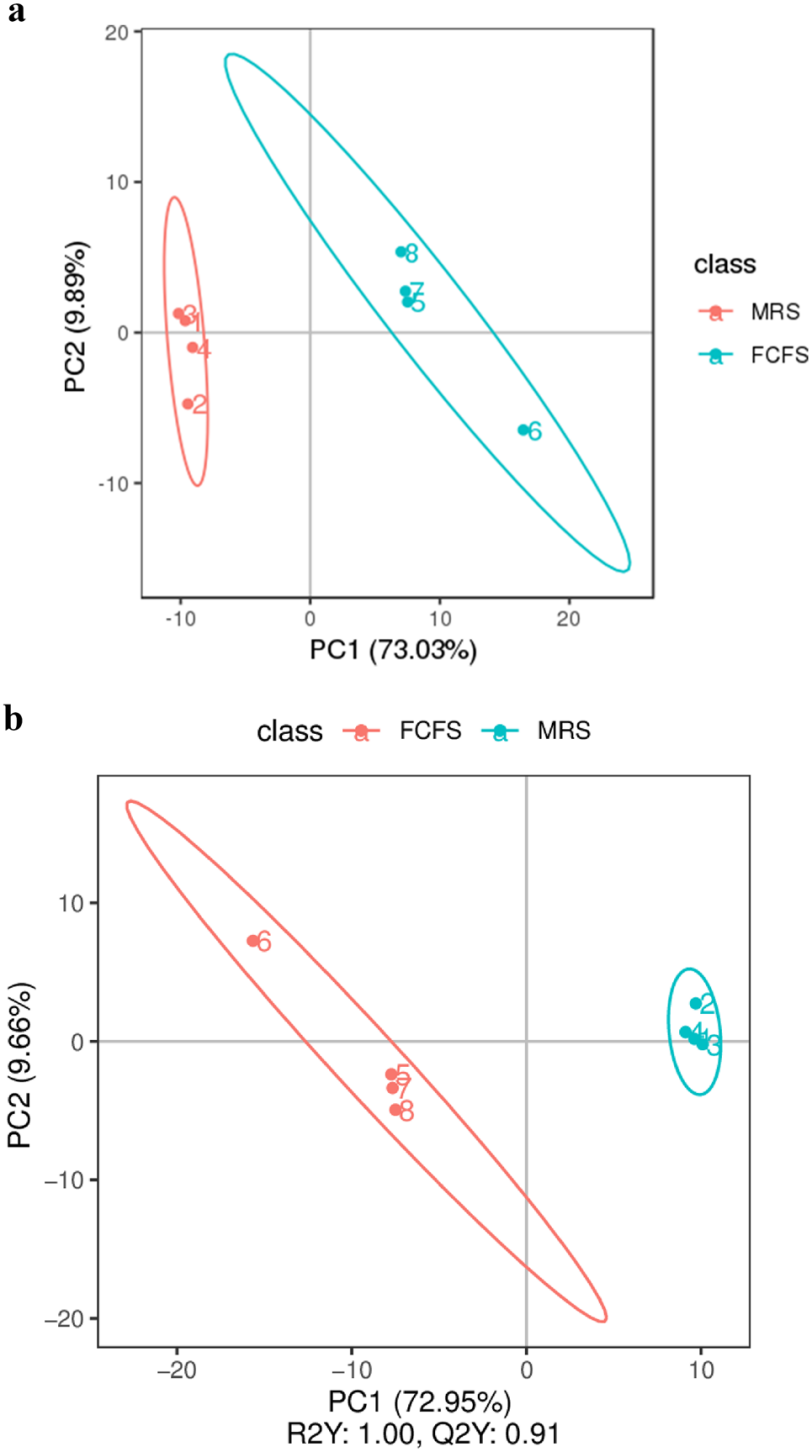
PCA plots (**a**) and OPLS-DA plots (**b**) of FCFS and MRS Differential metabolites. The abscissa PC1 and ordinate PC2 in the PCA plot represent the scores of the principal components at levels 1 and 2, respectively. The abscissa in the OPLS-DA plot is the score of the sample on the first principal component. The ordinate is the score of the sample on the second principal component. R2Y represents the explanatory rate of the model, and Q2Y is used to evaluate the predictive ability of the PLS-DA model.

In this study, the FCFS group and MRS groups were selected as the research subjects, with the differential metabolites between them analyzed using LC-MC. PCA analysis and OPLS-DA were employed to identify metabolites that contribute to the main differences between the two groups. As indicated in Fig. 9, the first principal component (PC1) was 82% of the variance, while the second principal component (PC2) was 9.89%. The Fig. 9 demonstrates a clear separation trend between the samples of FCFS and MRS Groups, with a cumulative contribution rate of 91.89% from the principal components. This suggests significant differences in metabolic profiles between FCFS and MRS groups. The OPLS-DA model was utilized to further investigate the metabolite differences. Figure 9 shows that the FCFS and MRS groups are completely separated in the model, R2Y represents the interpretation rate of the model, Q2Y is used to evaluate the predictive ability of the PLS-DA model, and the results show that R2Y is 1 and Q2Y is 0.91. R2Y > Q2Y and all close to 1, indicated that the model was well established and had good accuracy, and the differential metabolites could be screened by variable projection importance (VIP) method.

#### Volcano map and KEGG pathway bubble map analysis

**Figure 10:**
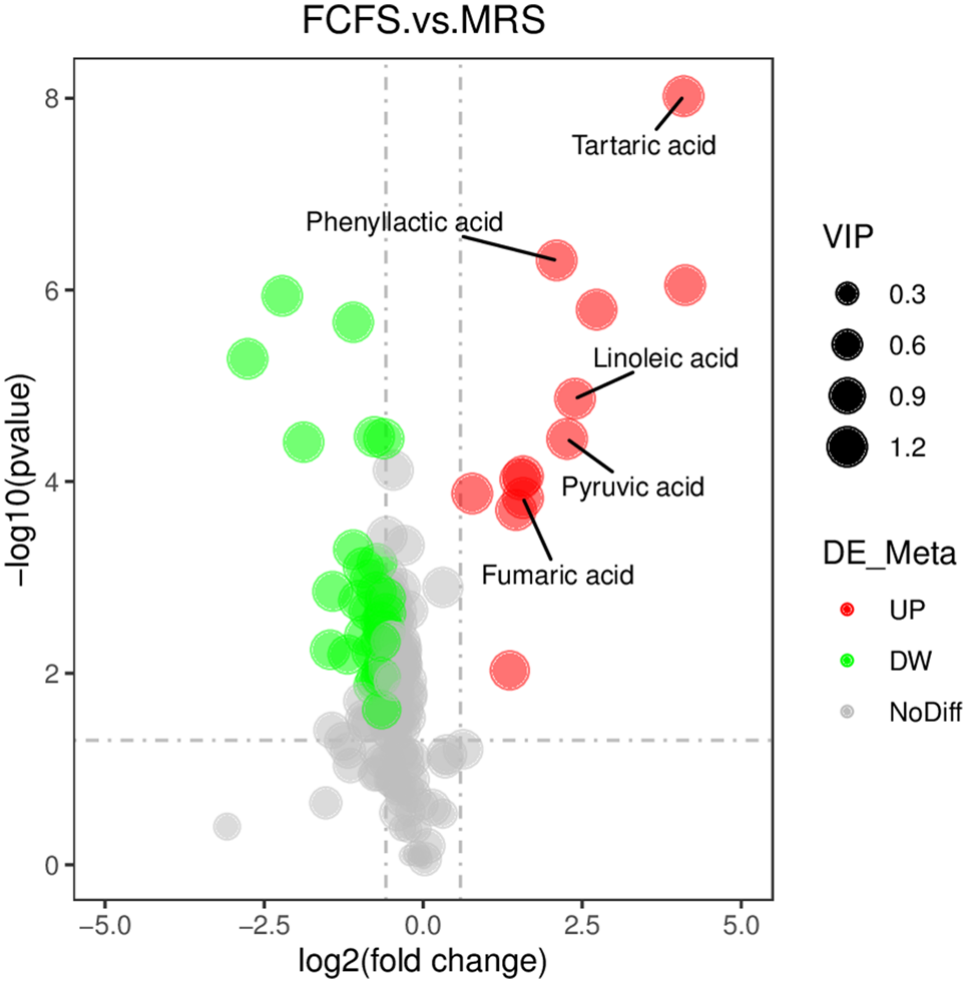
Volcano plot of FCFS and MRS Differential metabolites. The horizontal axis represents the fold change of the metabolites in different groups (log2FC), and the vertical axis represents the significance level of the difference (− log10(P-value)).

**Figure 11:**
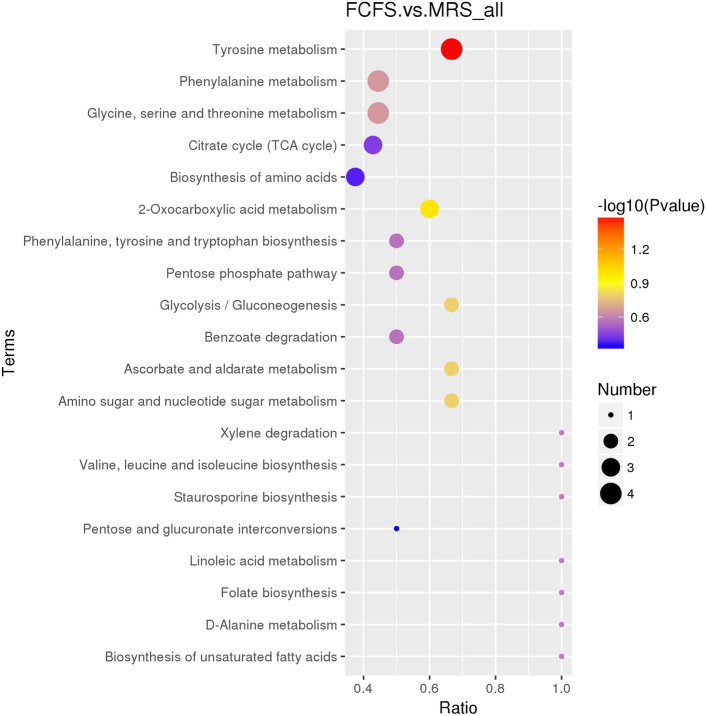
Bubble map of the effects of KEGG metabolic pathways in FCFS and MRS. In the KEGG pathway map^55–57^, the circles represent metabolites, in which the green solid circles are labeled as annotated metabolites, the red circles are up-regulated differential metabolites, the blue circles are down-regulated differential metabolites, and the yellow circles contain both up-regulated and down-regulated differential metabolites.

The volcano plot, as depicted in Fig. 10, visualizes the differential metabolites where red dots indicate significantly up-regulated substances, green dots indicate significantly down-regulated substances, and grey dots represent substances that are not significantly different between the two groups. The five most prominent metabolites identified were Fumaric acid, Tartaric acid, Phenyl lactic acid, Linoleic acid and Phenylpyruvic acid. Among these, the first four were all significantly up-regulated, while Phenylpyruvic acid was significantly down-regulated. Following the identification of 46 metabolites, they were annotated against the KEGG database to map their associated metabolic pathways, and the top 20 annotated metabolic pathways are shown in Fig. 11. The top five pathways with the highest enrichment of differential metabolites included Tyrosine metabolism, Phenylalanine metabolism Glycine, serine and threonine metabolism, Citrate cycle (TCA cycle) and 2-Oxcarboxylic acid metabolism.

## Discussion

Probiotic LAB have been demonstrated to exert positive effects on the anti-oxidative^10^, anti-inflammatory^34^, and anticancer activities^35^. The antioxidant capabilities of probiotics have been evaluated through IC^26^, CFS^10^ and CFE or their metabolites in vitro^36^. Previous research has indicated that strains such as *L. acidophilus*^32^, *L. casei*^37^ and *L. reuteri*^38^, possess antioxidative properties. Both IC and CFE of *S. thermophilus* and *Bifidobacterium longum*^32^, *L. casei*^39^ have demonstrated the capacity to scavengers hydroxyl radicals and inhibit linoleic acid peroxidation. Similarly, the CFS of *L. rhamnosus* has shown DPPH RSA, which is in line with our findings and recent research indicating. In our study, we assessed the antioxidant properties of the four LAB samples in vitro. We found that the CFS, IC and CFE all exhibited antioxidant capabilities, but the antioxidant properties of the four strains of LAB were found to vary. FCFS showed superior antioxidant activity compared to IC and CFE of the strain FGM, with higher DPPH RSA, hydroxyl RSA and linoleic acid peroxidation inhibitory activity. Overall, compared with other strains, FCFS had the best Linoleic acid peroxidation inhibitory activity, DPPH RSA was higher than that of *L. acidophilus* and *L. reuteri*, and Hydroxyl RSA was higher than that of *L. casei*.

Macrophages play an important role not only in immune responses but also in phagocytosis and tissue repair during damage or transitional inflammatory responses caused by microbial infections^40^. Studies have shown that LPS stimulates macrophages to induce oxidative stress by activating inducible nitric oxide synthase (iNOS). Vitamin C may protect macrophages from LPS-induced oxidative stress by maintaining iNOS activity by increasing the stability of tetrahydrobiopterin^41^. In this study, 5 μL, 25 μL, 50 μL and 125μL FCFS was applied to RAW264.7 cells. It was found that the FCFS promoted cell proliferation and there was no significant effect of each dose on ROS levels in RAW 264.7 cells, indicating that FCFS did not cause intracellular ROS disturbances. However, there was a dose-dependent relationship between the NO production in RAW 264.7 cells and FCFS. Studies have shown that NO is lipid-soluble, it can affect the production of several cytokines and enhance the function of the immune system^42^. SOD and GSH-Px are considered the main antioxidant enzymes for the elimination of ROS in vivo^43^. It was also found that 25 μL and 50 μL FCFS could increase the SOD activity of RAW264.7 cells, but only 25μL FCFS had a significant effect on the GSH-Px activity. When the concentration of FCFS was increased to 125 μL, SOD and GSH-Px activities in RAW 264.7 cells were reduced. Therefore, 25 μL and 125 μL FCFS were used for subsequent experiments. In this study, 0.5 μg/mL LPS treatment at 37 ℃ for 24 h successfully induced a significant increase in cell viability in RAW264.7 cells. They were treated with 25 μL and 125 μL FCFS for 12 h in RAW264.7 cells and then stimulated with LPS for 24 h to evaluate the preventive and protective effects of FCFS on oxidative stress in RAW 264.7 cells. The results showed that 25 μL FCFS could significantly relieve the stress on SOD, GSH-Px activities and NO content in LPS induced RAW 264.7 cells.

To further investigate the potential mechanisms of the antioxidant properties of the strain FGM, we examined the expression profiles of metabolite levels in the FCFS, performing correlation analyses to better understand specific metabolites. Due to the differences in metabolites, and the fact that antioxidant active components occurring differently in each strain, the metabolome has become an effective tool for analyzing metabolite accumulation patterns and has been used for nutritional analysis of many substances^44^. In the quantitative N300 assay performed on the FCFS, a total of 156 metabolites were detected, and 46 metabolites (12 up-regulated and 34 down-regulated) among them were significantly different. Further, the 12 up-regulated metabolites were tartaric acid, phenyl lactic acid, lactic acid, indole lactic acid, linoleic acid, pyruvic acid, maleic acid, homovanillic acid, xylose, fumaric acid, glyceraldehyde, hydroxyphenyl lactic acid which had been produced during the fermentation of the *S. alactolyticus* strain FGM. Among them, lactic acid, fumaric acid, indole lactic acid, linoleic acid and pyruvic acid (FC value > 4) were the main products. Most of the up-regulated metabolites have been reported with antioxidant properties, such as tartaric acid^45^, phenyl lactic acid^46^, lactic acid^47^, linoleic acid^48^, pyruvic acid^49^, maleic acid^50^, homovanillic acid^37^, xylose^51^, fumaric acid^52^, glyceraldehyde^53^, hydroxyphenyl lactic acid^54^.

## Conclusion

In the present study, the CFS, IC and CFE from *S. alactolyticus* strain FGM were all capable of DPPH RSA, hydroxyl RSA and inhibiting linoleic acid peroxidation. Among these, the FCFS showed then strongest scavenging ability. Beyond its antioxidant physicochemical properties, FCFS was also showed antioxidant activity through the enhancement of SOD and GSH-Px activities, as well as a regulatory effect on NO content in LPS induced RAW 264.7 cells. The antioxidant activity exhibited by FCFS were possibly related to tartaric acid, phenyl lactic acid, lactic acid, linoleic acid, pyruvic acid, maleic acid, homovanillic acid, xylose, fumaric acid, glyceraldehyde and hydroxyphenyl lactic acid, which are secreted by *S. alactolyticus* strain FGM. This study provides new evidence for the practical applications of the *S. alactolyticus* strain in livestock and poultry breeding in the future.

### Supplementary Information

Supplementary Information.
